# Editorial: Bridging the knowledge gap: enhancing research on women's participation in sports

**DOI:** 10.3389/fspor.2026.1953828

**Published:** 2026-08-24

**Authors:** J. M. Schulz, E. Martinez-Rosales

**Affiliations:** 1 Department of Physical Therapy, Faculty of Medicine, University of British Columbia, Vancouver, BC, Canada; 2 Arthritis Research Canada, Vancouver, BC, Canada; 3 La Trobe Sport and Exercise Medicine Research Centre, School of Allied Health Human Services and Sport, La Trobe University, Melbourne, VIC, Australia; 4 Department of Physiology, Faculty of Pharmacy, Sport and Health University Research Institute (iMUDS), University of Granada, Granada, Spain

**Keywords:** female athlete, female athlete health, physical activity, sport participation, women's health, women's sport

Women's sport is experiencing unprecedented growth, with increasing participation, enhanced visibility, and expanding opportunities for athletes worldwide. As more women and girls engage in sport across the lifespan, it is crucial that the scientific evidence needed to support their health, performance, and long-term participation keeps pace. However, because they remain under-represented in sport research, there are significant gaps in evidence to guide their health, performance, and participation (1, 2). While this Research Topic initially sought to better understand women's participation in sport, the breadth of submissions underscores a broader message: fostering participation requires research that supports females/women/girls throughout their lifespan and sporting journeys. Spanning physiology, injury prevention, psychosocial influences, and inclusive sporting environments, these studies highlight the diverse factors that enable females/women/girls not only to participate, but to thrive in sport and physical activity (Figure 1).

**Figure 1:**
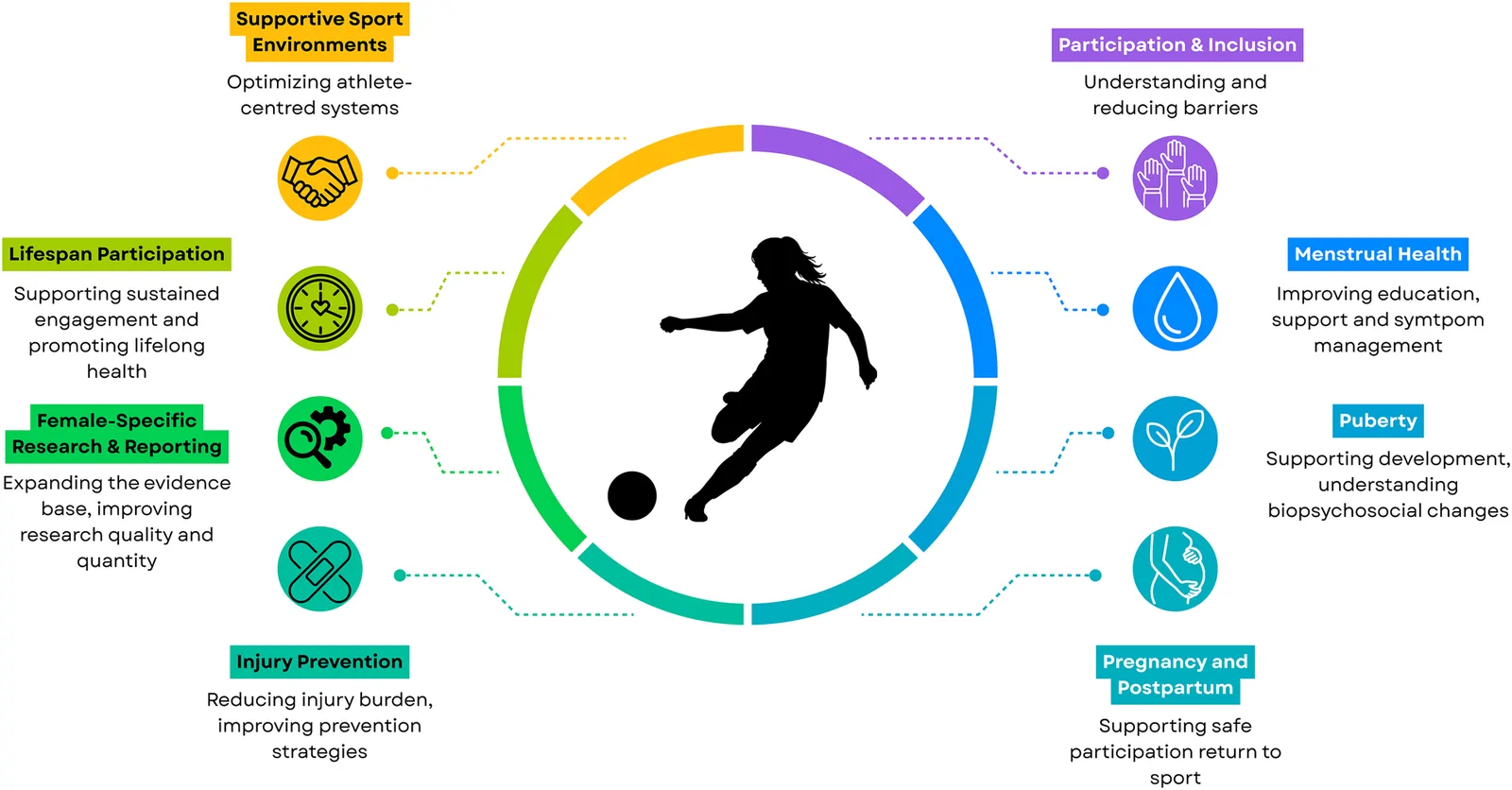
Common themes emerging from this special topic.

## The articles in this special issue can broadly be categorized into three overarching themes

1. Understanding participation across the lifespan

Participation in sport is shaped by a complex interplay of individual, social, and environmental factors that evolve across the lifespan. Several studies in this Research Topic explored the barriers and facilitators influencing engagement in sport and physical activity at different life stages. Among adolescents, sports participation was associated with greater body appreciation, reinforcing the potential for sport to positively influence psychological well-being (Meyers et al.). In young adults, perceived barriers to physical activity extended beyond individual motivation and included harassment, body image concerns, lack of social support, safety, weather, and the absence of same-gender role models (Prieto-González and Alkouatli). Research also emphasized the importance of creating inclusive environments, including the development of qualitative approaches to better understand how physical activity in community and urban spaces can foster the social inclusion of immigrant women (Blanco-Ayalaet al.) For older women, participation was further influenced by professional demands, caregiving responsibilities, and persistent gendered expectations surrounding aging and physical activity (Van Wyk et al.). Taken together, these findings emphasize that increasing women's participation is not simply a matter of expanding opportunities, it also requires addressing the social, cultural, and structural barriers that shape the experiences of women and girls throughout the lifespan.

2. Female athlete health as a foundation for participation

Female athletes have specific biological, sociocultural and environmental considerations that can impact sport participation and health outcomes (3). Several studies in this Research Topic highlighted female-specific health considerations across different life stages, ranging from menstrual health and puberty to pregnancy and postpartum return to sport. Collectively, studies exploring menstrual health identified themes of significant impact on training and competition, uncertainty surrounding management strategies, and a desire for more supportive sporting environments (Chica-Latorre et al.; Ryman Augustsson and Findhé-Malenica; Ambrosio and Adam). Similarly, female athletes reported limited perceived knowledge and high knowledge need of puberty-related biopsychosocial changes, highlighting opportunities to improve education and support during this critical developmental period (Radovan et al.) Among athlete-mothers, many continued training throughout pregnancy; however, participants frequently described inadequate guidance from healthcare professionals, limited childcare support, and challenges balancing breastfeeding with training upon return to sport (Hewitt et al.) What emerges across these studies is a shared need for deeper understanding of female-specific health across the lifespan, paired with education, clinical care, and sporting environments that address individual needs.

3. Optimizing performance and reducing injury

Performance optimization requires evidence generated in female athletes rather than extrapolating from male cohorts. The studies in this Research Topic contribute to this growing evidence base across a range of sporting contexts. A systematic review identified a substantial risk of musculoskeletal injury in collegiate cheerleading, and pointed to inconsistent injury definitions and underreporting as key barriers to more reliable surveillance (Haymeset al.) Similarly, a scoping review in female trail runners highlighted links between nutrition, body composition and performance outcomes, however; female-specific variables such as menstrual cycle status and hormonal contraceptive use were infrequently reported (Espasa-Labrador et al.). Addressing a related performance and recovery concern, pelvic compression garments improve running biomechanics and perceived pelvic floor support among postpartum runners with pelvic floor dysfunction (Donnelly et al.). In football, studies suggested that while heading during a typical match did not impair balance, it may influence fine motor control, (Palmer et al.) and that although injury incidence did not differ between menstrual bleeding and non-bleeding phases, injuries sustained during menstruation may be associated with a greater injury burden (Ferrer et al.). These studies emphasize the need for standardized reporting, greater attention to female-specific physiological factors, and sustained investment in specific research necessary to strengthen injury prevention, performance optimization, and return-to-sport strategies.

The continued growth and visibility of women's sport marks a pivotal shift in the sporting landscape and underscores the need for research that reflects their diverse experiences. The articles in this Research Topic illustrate that supporting participation requires more than increasing opportunities, it requires research that recognizes the unique physiological, psychological, and social factors influencing health and performance throughout the lifespan. By addressing these complexities, researchers, clinicians, coaches, and sporting organizations can work together to create environments in which women are empowered to participate, perform, and remain engaged in sport at every stage of life.
